# An Evaluation of the Cellular and Humoral Response of a Multi-Epitope Vaccine Candidate Against COVID-19 with Different Alum Adjuvants

**DOI:** 10.3390/pathogens13121081

**Published:** 2024-12-09

**Authors:** Lineth Juliana Vega Rojas, Rocío Alejandra Ruíz-Manzano, Miguel Andrés Velasco-Elizondo, María Antonieta Carbajo-Mata, Diego Josimar Hernández-Silva, Mariana Rocha-Solache, Jesús Hernández, Rosa Martha Pérez-Serrano, Guadalupe Zaldívar-Lelo de Larrea, Teresa García-Gasca, Juan Mosqueda

**Affiliations:** 1 Immunology and Vaccines Laboratory, Facultad de Ciencias Naturales, Universidad Autónoma de Querétaro, Campus Aeropuerto, Carretera a Chichimequillas, Ejido Bolaños, Querétaro 76140, Mexico; lineth.vega@uaq.mx (L.J.V.R.); ros-ale2@hotmail.com (R.A.R.-M.); mike.and13@hotmail.com (M.A.V.-E.); diego.hernandez@uaq.mx (D.J.H.-S.); mrocha02@alumnos.uaq.mx (M.R.-S.); 2 Consejo Nacional de Humanidades, Ciencias y Tecnologías (CONAHCYT), Av. Insurgentes Sur 1582, Alcaldía Benito Juárez, Crédito Constructor, Ciudad de México 03940, Mexico; 3 Instituto de Neurobiología UNAM, Laboratorio Universitario del Bioterio, Universidad Nacional Autónoma de México, Querétaro 76230, Mexico; 4 Laboratorio de Inmunología, Centro de Investigación en Alimentación y Desarrollo, A.C, Hermosillo 83304, Mexico; jhdez@ciad.mx; 5 Advanced Biomedical Research Center, School of Medicine, Universidad Autónoma de Querétaro, Querétaro 76176, Mexico; rosa.perez@uaq.mx (R.M.P.-S.); guadalupe.zaldivar@uaq.mx (G.Z.-L.d.L.); 6 Facultad de Ciencias Naturales, Universidad Autónoma de Querétaro, Av. de las Ciencias s/n, Juriquilla, Querétaro 76230, Mexico

**Keywords:** chimeric vaccine, COVID-19, SARS-CoV-2, recombinant proteins, antiviral response, neutralizing antibodies, cellular response

## Abstract

SARS-CoV-2 (*Betacoronavirus pandemicum*) is responsible for the disease identified by the World Health Organization (WHO) as COVID-19. We designed “CHIVAX 2.1”, a multi-epitope vaccine, containing ten immunogenic peptides with conserved B-cell and T-cell epitopes in the receceptor binding domain (RBD) sequences of different SARS-CoV-2 variants of concern (VoCs). We evaluated the immune response of mice immunized with 20 or 60 µg of the chimeric protein with two different alum adjuvants (Alhydrogel^®^ and Adju-Phos^®^), plus PHAD^®^, in a two-immunization regimen (0 and 21 days). Serum samples were collected on days 0, 21, 31, and 72 post first immunization, with antibody titers determined by indirect ELISA, while lymphoproliferation assays and cytokine production were evaluated by flow cytometry. The presence of neutralizing antibodies was assessed by surrogate neutralization assays. Higher titers of total IgG, IgG_1_, and IgG_2a_ antibodies, as well as increased proliferation rates of specific CD4^+^ and CD8^+^ T cells, were observed in mice immunized with 60 μg of protein plus Adju-Phos^®^/PHAD^®^. This formulation also generated the highest levels of TNF-α and IFN-γ, in addition to the presence of neutralizing antibodies against Delta and Omicron VoC. These findings indicate the potential of this chimeric multi-epitope vaccine with combined adjuvants as a promising platform against viral infections, eliciting a T_H1_ or T_H1_:T_H2_ balanced cell response.

## 1. Introduction

Vaccination represents a significant contribution in immunology and medicine in the battle against illness and global epidemics [1,2]. It was key in eradicating smallpox and controlling rabies, influenza, tetanus, diphtheria, poliomyelitis, HPV (human papillomavirus), and COVID-19, supported by advancements in immunology, biotechnology, molecular biology, and biomedical development [3,4,5]. Currently, several platforms are being utilized to develop inactivated virus vaccines, viral vector vaccines (adenovirus), recombinant protein vaccines, live attenuated vaccines, and peptide vaccines [6,7,8].

Chimeric vaccines are modern strategies that use immuno-bioinformatic tools for the identification and selection of various immunogenic peptides as antigens. These vaccines comprise a wide range of epitopes, including both B-cell epitopes and T-cell epitopes, and those presented by MHC class I and MHC class II molecules. This approach enables the recognition of various epitopes by distinct B-cell receptors (BCRs) and T-cell receptors (TCRs), capable of detecting multiple variants of a single pathogen or even different pathogens simultaneously [9,10,11,12,13]. Nevertheless, without adjuvants, chimeric vaccines are generally poorly immunogenic; thus, adjuvants are required to enhance immunogenicity, particularly the duration and intensity of the immune response [1,2,3].

A century ago, the formulation of human vaccines with adjuvants was legalized for the first time, leading to the more common use of adjuvants. Aluminum adjuvants, such as aluminum hydroxide (Alhydrogel^®^) and aluminum phosphate (Adju-Phos^®^), are widely used in human vaccines against various illnesses [4,14]. They are considered the “gold standard” of adjuvants licensed for human vaccines in the USA and/or Europe [15,16].

The initial outbreak of COVID-19 was caused by an infection with the emerging Severe Acute Respiratory Syndrome Coronavirus 2 (SARS-CoV-2, *Betacoronavirus pandemicum*), which started in the city of Wuhan, China, and became an unprecedented global crisis. The virus’s accelerated transmission to most countries and its high lethality led the World Health Organization (WHO) to declare the situation an international emergency on January 30, 2020, a month after the first case was reported on December 31, 2019. Eventually, on March 11, it was officially declared a pandemic [7,8].

Vaccine candidates against SARS-CoV-2 were developed relatively quickly, with different alternatives for design and production utilized for global deployment [8,17]. The formulation of vaccines against SARS-CoV-2 is crucial to generate an antiviral response, chiefly characterized by the induction of T helper 1 (T_H1_) lymphocytes and the secretion of TNF-α and IFN-γ [18]. It is understood that the antigen and adjuvant’s nature drives the humoral and cellular immune response [8,19]. In this context, alum adjuvants trigger a local inflammatory response by releasing alarmins (e.g., IL-33) through cellular necrosis. This action induces and orchestrates a type 2 response characterized by the recruitment and expansion of innate lymphoid cells 2 (ILC-2), T helper 2 (T_H2_) lymphocytes, B- and plasma cells, along with antibodies and the secretion of IL-4, IL-5, IL-13, IL-6, and TNF-α. The latter two are produced by inflammasome activation via the NLPR3 receptor. Hence, a cellular response that activates CD4^+^ and subsequently CD8^+^ lymphocytes (T_H1_) is absent [20,21,22].

Despite the protective humoral response stimulated by alum adjuvants linked to vaccine antigens for viral diseases, it is also crucial to elicit a response capable of countering viral infections. Consequently, incorporating additional molecules to generate a T_H1_-based immune response crucial against viral antigens is necessary. Within this context, the addition of lipophilic adjuvants, including AS04, MF59, AS01, and monophosphoryl lipid A (MPL, PHAD) into formulations with alum adjuvants, offers a promising strategy. These adjuvants, potent immunomodulators and highly effective delivery devices for protein antigens, interact with Toll-like receptor 4 (TLR4) to promote T_H1_ cell activation. The result is an enhancement of the immune response [16,23]. MPL stimulates a type 1 cell response and contributes to a balanced T_H1_:T_H2_ ratio. The synergistic action mechanisms demonstrated by these adjuvants and targeted SARS-CoV-2 antigens aim to stimulate a protective response against COVID-19 [6,24]. Therefore, this study’s objective was to assess the humoral and cellular responses from two aluminum adjuvants in various formulations with and without PHAD^®^, using a multi-epitope vaccine against COVID-19 within a comprehensive immunization scheme.

## 2. Materials and Methods

### 2.1. Peptide Selection and Chimeric Protein Design for the SARS-CoV-2 Spike Protein

The amino acid sequences of the SARS-CoV-2 variants from the National Center for Biotechnology Information (NCBI) database (published on 6 April 2020) were used to identify conserved and semiconserved immunogenic peptides and to design a chimeric protein. The spike protein sequences were subjected to the analyses described in previously published studies [12]. Thereafter, conserved and semiconserved peptides in the RBD sequences of different SARS-CoV-2 variants such as Alpha, Beta, Gamma, Delta, and Omicron were identified through multiple sequence alignment using MUSCLE (https://www.ebi.ac.uk/Tools/msa/muscle/ accessed on 20 July 2021). Subsequently, B- and T-cell epitope predictions for the conserved peptides were carried out using immune bioinformatics tools [12].

**Figure 1:**
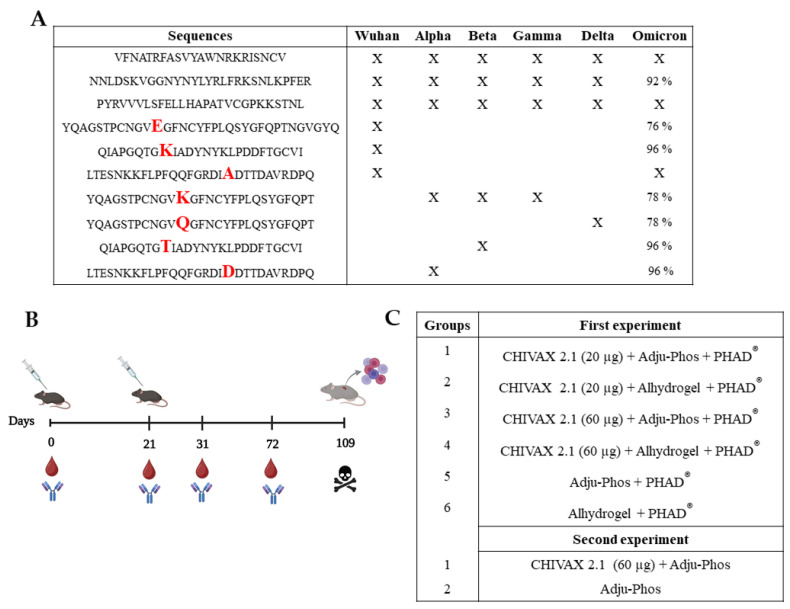
Experimental design. (**A**) A list of peptides used in the multi-epitopic protein CHIVAX 2.1 design and the presence of each of their variants (VoCs). Some peptides are 100% conserved in all the spike protein sequences (in Wuhan’s, XBB1; Alpha, B.1.1.7; Gamma, B.1.1.248; and Delta, B.1617.2) and some peptides are 100% conserved in a single variant. A similarity of 100% is represented by an X. In the case of the Omicron variant (BA.1.25), the percentage of similarity is added for each peptide sequence. Letters in red represent the amino acid substitutions in the peptide sequences. (**B**) Experimental design: Two subcutaneous immunizations (SC) are given on days 0 and 21. Serum samples are obtained on days 0, 21, 31, and 72. The animals in some groups are humanely euthanized on day 109. Cellular immune responses are analyzed on day 109 by culturing splenocytes. (**C**) Groups of animals are immunized with multi-epitopic protein (CHIVAX 2.1) in two independent experiments with and without PHAD^®^. The first experiment is performed using two concentrations of CHIVAX 2.1 and two alum adjuvants plus PHAD^®^, while the second experiment is carried out with 60 µg CHIVAX 2.1 and only one alum adjuvant.

The amino acid sequence of the S protein was analyzed with bioinformatics tools, such as ABCPred, to predict peptides that are T- or B-cell epitopes. Predicted epitopes, conserved in the variants B.1.1.7, B.1.351, P.1, B.1.525, B.1.429, P.2, A.23.1, B.1.617.1, Delta, and Omicron, were integrated into a protein assembly using the Gentle Software 1.9.4 (Magnus Manske, the University of Cologne, Köln, Germany). The multi-epitope recombinant protein CHIVAX 2.1 was designed by selecting conserved peptides from the original Wuhan strain and from the variants of concern (VoCs), as depicted in Figure 1A. Tandem repetitions of the sequences were used in the design of the chimeric protein to increase the antigen’s molecular weight, thereby enhancing its immunogenicity. The amino acid sequences for the VoC exhibited a 76 to 100% conservation level. Once each immunogenic peptide was chosen, a multi-epitopic sequence was structured such that the peptides were organized into a single amino acid primary structure, aligning with the order in the RBD sequence as previously described, without linkers [12]. To optimize the chimeric protein expression in the bacterial model, the preferential codons for the BL21 AI strain of Escherichia coli were improved using the Codon Optimization OnLine tool [25], along with a calculation of the isoelectric point and the total average hydrophobicity.

The nucleotide sequence was commercially acquired from Bio Basic Inc., Markham, ON, Canada. The DNA sequence of the chimeric gene was cloned and inserted into the PET30 (A) vector, with the process conducted by GenScript, Piscataway, NJ, USA. Additionally, individual peptides were commercially synthesized in both linear and 8-branch multiantigen peptide (MAP-8) formats by Peptide2.0 Inc., Chantilly, VA, USA.

### 2.2. Bioethics Protocols, Animals, and Immunization Experiments

All animal procedures in this study were approved by the Research Ethics Committee of the Universidad Autónoma de Querétaro (DIP/291-2020). Female C57-BL/6J mice (9–10 weeks old) were purchased from the Universidad Nacional Autónoma de México (UNAM-INB). The animals were housed in cages and provided with food and water (Conejina T, Purina, St. Louis, MO, USA) ad libitum under a 12:12 h light–dark cycle at 21 ± 2 °C with a relative humidity of 40–60%. The mice were acclimated for one week and subsequently inoculated subcutaneously twice (on days 0 and 21) with 300 µL of the vaccine (Figure 1B). Recombinant protein (20 and 60 µg) was formulated with 80 µg of Al^3+^/50 µL of Adju-Phos^®^ or 85% Alhydrogel^®^, respectively (Croda, Snaith, East Cowick, UK), and 20 µg/dose of 3D-(6-acyl) PHAD^®^ (Avanti^®^ Polar Lipids, Inc., Alabaster, AL, USA). All vaccine formulations were dissolved in NaCl (pH 7) and sonicated (94539, Biobase, Osgood Common, Fremont, CA, USA) at 150 W, an amplitude of 80%, and a pulse of 9 s/1 s.

Two-immunization regimens were implemented, wherein the animals were randomly assigned into six groups (*n* = 5) for the initial experiment: (1) CHIVAX 2.1 (20 µg) + Adju-Phos + PHAD^®^, (2) CHIVAX 2.1 (20 µg) + Alhydrogel + PHAD^®^, (3) CHIVAX 2.1 (60 µg) + Adju-Phos + PHAD^®^, (4) CHIVAX 2.1 (60 µg) + Alhydrogel + PHAD^®^, (5) Adju-Phos + PHAD^®^ and (6) Alhydrogel + PHAD^®^. Following the results from the first experiment, two more animal groups were immunized with 1) CHIVAX 2.1 (60 µg) + Adju-Phos and 2) Adju-Phos (Figure 1C). Preimmune and postimmune blood drawn from the lateral saphenous vein happened at 0, 21, 31, and 72 days. The blood was collected in BD Microtainer tubes, centrifuged (1500× *g* for 15 min) to separate the serum, and afterward stored at −80 °C. Animals were euthanized using carbon dioxide (CO2) inhalation and exsanguinated via a cardiac puncture on day 109.

### 2.3. Serum-Specific Antibodies by ELISA

An indirect ELISA was conducted to evaluate the humoral immune response to the chimeric protein vaccine (CHIVAX 2.1). ELISA plates (flat-bottom polystyrene high-binding microplates, Corning, NY, USA) were coated with 100 µL of CHIVAX 2.1 (2.5 µg/mL) in a carbonate buffer (pH 9.6) and left overnight at 4 °C. The plates were subsequently washed three times (200 µL/well) with phosphate-buffered saline containing 0.05% Tween-20 (PBS-T) at room temperature (25 ± 1 °C). The plates were blocked with 200 µL/well of 5% low-fat milk suspended in PBS-T (pH 7.4) for 1 h at 37 °C and 200 rpm. After three additional washings, serum from days 0, 21, 31, and 72 post immunization (primary antibody, 100 µL/well) in serial dilutions from 1:200 to 1:51,200 was added and incubated (37 °C, 1 h and 200 rpm). Plates were then washed with PBS-T to eliminate unbound antibodies. Horseradish peroxidase-conjugated anti-mouse IgG, H + L (115035145 Jackson ImmunoResearch, Baltimore, MD, USA), IgM, H + L (ab97250 Abcam, Trumpington, Cam, UK), IgG_2b_, H + L (115235207 Jackson ImmunoResearch, Baltimore, MD, USA), IgG_2a_, H + L (ab97245 Abcam, Trumpington, Cam, UK), and IgG_1_, H + L (ab97240 Abcam, Trumpington, Cam, UK) were employed. Secondary antibodies were diluted 1:6000 with PBS and 2% skim milk (100 µL/well), further incubated at 37 °C for 1 h at 200 rpm, and washed three times (100 µL/well). O-phenylenediamine dihydrochloride (0.4 mg/mL; Sigma-Aldrich, St. Louis, MO, USA) suspended in sodium citrate and citric acid buffer with 4 µL H_2_O_2_ (30% *v*/*v*) served as the substrate. Swine serum from a previous immunization experiment was used as both the positive and negative control [12]. Finally, absorbance was measured in an ELISA plate reader (Microplate Absorbance Reader, Bio-Rad, Hercules, CA, USA) at 450 nm after 20 min. MPM 6.exe software 3.2 (Bio-Rad) was used to analyze the results, with each sample analyzed in triplicate. The results are expressed as the mean ± standard deviation (SD).

### 2.4. SARS-CoV-2 Surrogate Neutralization Assay

The assay was performed as previously described [26], with minor modifications. We used Maxisorp ELISA 96-well microwell plates (Thermo Fisher Scientific, Waltham, MA, USA). Each well was coated with 100 µL of the recombinant RBD of the following SARS-CoV-2 variants, Delta, B.1.617.2, and Omicron B.A.1, at a concentration of 2 µg/mL. They were incubated overnight at 4 °C in 100 nM carbonate–bicarbonate buffer (pH 9.5), then washed and blocked with PBS + 10% FCS. The serum, diluted to 1:20, was incubated for 1 h at 25 °C. After washing, hACE_2_–mouse Fc [27] was added at a constant saturating concentration (160 nM) and maintained for 1 h at 25 °C. After further washing, bound hACE_2_ was detected with a goat anti-mouse IgG coupled to alkaline phosphatase (dilution 1:500, Southern Biotech, Birmingham, AL, USA). ELISA plates were measured with Gen5 version 1.11.5 software (BioTek Instruments). The data were analyzed using GraphPad Prism version 8.4.2. We included one antibody-positive control (pooled from SARS-CoV-2-infected individuals) and one SARS-CoV-2 negative control (pooled human sera collected from individuals before the year 2019) in each plate. Each analysis was performed in triplicate. The results were expressed as a percentage of inhibition, calculated as (1 − (absorbance of sample/absorbance positive for ACE_2_)) × 100. Samples with 30% inhibition were considered positive [26,28].

### 2.5. Mouse Splenocyte Isolation

Splenocytes from animals administered with CHIVAX 2.1 (60 µg), + Adju-Phos + PHAD^®^, were obtained from immunized mice on day 109 after the first immunization. For this procedure, the spleen of each mouse was excised and mechanically disaggregated through a 50 µm nylon mesh, supplemented with RPMI-1640. Splenic erythrocytes were lysed with ACK buffer (Gibco, Grand Island, NY, USA) for 5 min at room temperature, centrifuged (1500 rpm/5 min/4 °C), and subsequently resuspended in PBS. Cell counts were conducted before and after CFSE labeling.

Mouse splenocytes were cultured in RPMI-1640, supplemented with 10% heat-inactivated fetal bovine serum (FBS) (Biowest, Lakewood Ranch, FL, USA), 200 mM L-glutamine (25030, Gibco, Grand Island, NY, USA), 1% penicillin/streptomycin, 0.2% sodium pyruvate, and 0.2% 2-mercaptoethanol (Sigma-Aldrich, St. Louis, MO, USA). The cells were maintained at 37 °C in a humidified atmosphere with 5% CO_2_.

### 2.6. Labeling for Cell Proliferation Assay

Labeling was conducted using the Trace^TM^ CFSE Cell Proliferation Kit (C34554, Invitrogen, Life Technologies, Carlsbad, CA, USA), in accordance with the manufacturer’s instructions. Briefly, splenocytes (1 × 10^6^ cells/well) were incubated with 5 µM CFSE (final concentration) at room temperature for 10 min. The labeling reaction was then quenched with four volumes of ice-cold PBS supplemented with 10% heat-inactivated FBS (Biowest, Lakewood Ranch, FL, USA). The suspension was subsequently centrifuged (1500 rpm/5 min/4 °C) and then resuspended in PBS. Lastly, the labeled splenocytes (2 × 10^5^ cells/well) were counted and seeded in 96-well plates.

### 2.7. In Vitro Stimulation

Cells were treated with CHIVAX 2.1 at concentrations of 5, 10, and 20 µg/mL. For the positive control, concanavalin A (ConA) (C5275, Sigma-Aldrich, St. Louis, MO, USA) was implemented at a concentration of 0.25 µg/mL to activate splenocytes. Untreated cells were used as the negative control. Each of these cell groups was cultured for 72 h at 37 °C in a humidified environment containing 5% CO_2_.

The cells were harvested and labeled with Zombie Dye Violet (423117, Biolegend, San Diego, CA, USA) for 20 min before being washed with FACS buffer (PBS, 2% FBS, 0.02% NaN_3_ for Fc blocking). Subsequently, approximately 5 × 10^5^ cells were incubated with anti-CD16/CD32 (Mouse BD Fc Block, 553142, BD Biosciences, San Jose, CA, USA) for 20 min at 4 °C. After incubation, the cells were washed and labeled with the following antibodies: APC-conjugated anti-CD3 (100236, Clone17A2, Biolegend), diluted at 1.25:100; PE-conjugated anti-CD8 (553032, Clone 53-6.7, BD Biosciences, San Jose, CA, USA), diluted at 1.25:100; BV510-conjugated anti-CD4 (100553, Clone RM4-5, Biolegend, San Diego, CA, USA), diluted at 0.625:100; and APC-Cy7-conjugated anti-CD19 (557655, Clone 1D3, BD Biosciences, San Jose, CA, USA), diluted at 0.625:100. The gating strategy is described in Supplementary Material (Figure S1).

The intracellular fluorescence intensity of CFSE within the cells was assessed using a BD FACSVerse flow cytometer (BD Biosciences, San Jose, CA, USA) in the FITC channel. The flow cytometer was calibrated with Facsuite CS&T Beads (650621, BD Bioscience, San Jose, CA, USA), and data were analyzed using FlowJo™ software 10.7 (Treestar Inc., Ashland, OR, USA). Compensation was assessed with the same FlowJo software using unstained samples and single-stain controls. Positive cells were identified by gating subsequent to unstained cells. All measurements were performed in duplicate, and the percentages of proliferative cells, along with the standard error means (SEMs) of the fold change relative to the control, are graphically presented.

### 2.8. Cytokine Analysis by Flow Cytometry

At 20 h post stimulation, 50 μL of the media supernatant was carefully extracted, centrifuged, and transferred to another tube. Cytokines were quantified using the BD CBA Mouse T_H1_/T_H2_/TH_17_ Cytokine Kit (560485, BD Biosciences, San Jose, CA, USA), which allows for the simultaneous detection of seven cytokines in the mice. The kit provides a mixture of seven capture beads designed to identify interleukin-2 (IL-2), interleukin-4 (IL-4), interferon-γ (IFN-γ), tumor necrosis factor (TNF), interleukin-17A (IL-17A), and interleukin-10 (IL-10) in a single sample. The Cytokine Kit Manual was followed for the assay procedure. Previously, the seven specific antibodies for each cytokine had been mixed with the capture beads. Subsequently, 50 μL of the unknown serum sample and standard dilutions, 70 μL of the mixed capture beads, and 70 μL of phycoerythrin (PE) detection reagent were added to each assay tube and incubated at room temperature in the dark for 2 h. Following this, 1 mL of wash buffer was added, with the mixture then centrifuged at 200× *g* for 5 min. Ultimately, the sample and standard dilution pellets were resuspended in 300 μL of wash buffer. The established concentrations for each cytokine were 0, 20, 40, 80, 156, 312.5, 625, 1250, 2500, and 5000 pg/mL. The measurement criteria consisted of 2500 events with the following parameters: forward scatter (FSC) at 262.6 V, side scatter (SSC) at 253.9 V, and both with a threshold of 10,000. The PE step had a voltage of 456.3, and the CBA Red step had a voltage of 292.1, both characteristic of a 5000 threshold. The samples were measured on a BD FACSVerse™ System flow cytometer. Additionally, BD FACSuite™ v1.0.5.3841 was utilized for data acquisition, and data analysis was carried out using FCAP Array^TM^ Software 3.0 (652099, BD Bioscience, San Jose, CA, USA). Prior to the use of the flow cytometer, it had been calibrated with Facsuite CS&T beads (650621, BD Bioscience, San Jose, CA, USA).

### 2.9. Statistical Analyses

Statistical analyses were performed using GraphPad Prism version 8. An ordinary two-way analysis of variance (ANOVA) with Holm-Sidak’s multiple-comparison test was utilized for comparisons among different groups. An unpaired Student’s *t*-test was also used between two vaccine formulations. The two-tailed nonparametric Mann-Whitney test was used to compare results between groups. An unpaired *t*-test was conducted to compare the proportion of negative control and treated cells and for the comparison of cytokines in the media supernatant. Following this, Welch’s correction was applied when Fisher’s test was conducted to make comparisions, showing significant differences in the variance. The data are presented as mean and error bars (SEM).

## 3. Results

### 3.1. Humoral Response Against SARS-CoV-2

**Figure 2:**
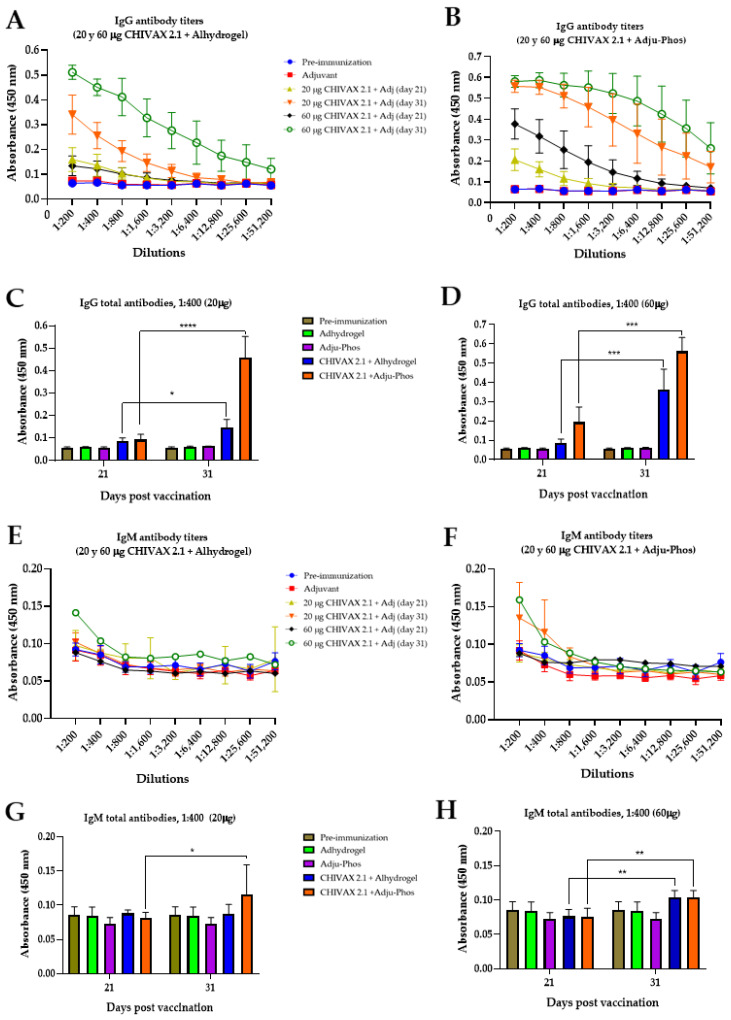
Serum IgG and IgM antibody production against two different concentrations of chimeric protein (CHIVAX 2.1) and two alum adjuvants plus PHAD^®.^ Animals were immunized two times (0 and 21 days) by the subcutaneous route. (**A**) Specific IgG antibody titers using Alhydrogel^®^. (**B**) Specific IgG antibody titers using Adju-Phos^®^. A ratio of 1:400 dilutions for (**C**) IgG for 20 µg vaccine and (**D**) IgG for 60 µg vaccine. (**E**) Specific IgM titers using Alhydrogel^®^, (**F**) specific IgM antibody titers using Adju-Phos^®^. A ratio of 1:400 dilutions for (**G**) IgM for 20 µg vaccine and (**H**) IgM for 60 µg vaccine. Each result corresponds to the mean value of 5 animals ± SD. Unpaired *t*-test with Holm–Sidak’s comparison between the same groups of immunized animals for different postvaccine times (days 21 and 31). The differences were considered significant: *p* ≤ 0.05 (*), *p* ≤ 0.001 (**), *p* ≤ 0.0001 (***), and *p* < 0.0001 (****).

The levels of serum-specific IgG and IgM antibodies on each immunization (0 and 21 days) against the multi-epitope vaccine “CHIVAX 2.1”—administered subcutaneously—were determined by an indirect ELISA. Figure 2A,B illustrate the IgG antibody responses in mice immunized with aluminum hydroxide (Alhydrogel^®^), aluminum phosphate (Adju-Phos^®^), and 20 µg or 60 µg doses of CHIVAX 2.1. Although all mouse groups immunized with CHIVAX 2.1 generated specific IgG antibodies, the response differed among formulations and was contingent on the adjuvant used. The maximum absorbance of IgG antibody production occurred 10 days post second immunization. Serum IgG antibody production was observed to be directly proportional to the CHIVAX 2.1 dosage. In contrast, the effect of adjuvants varied among the groups of immunized animals. On day 31 post first immunization, a decline in the absorbance of specific IgG serum production was observed when Alhydrogel^®^ was used (Figure 2A), while mice injected with Adju-Phos^®^ demonstrated consistent IgG levels with descending serum dilutions (Figure 2B). Total IgG for 20 µg and 60 µg of CHIVAX 2.1 treatments across various immunization groups (samples diluted 1:400) are represented in Figure 2C,D. Significant differences in total IgG antibody levels were detected for each adjuvant on days 21 and 31 post immunization with respect to vaccine concentration. Vaccines formulated with Adju-Phos^®^ demonstrated the best response to the adjuvant with CHIVAX 2.1.

The generation of IgM antibodies is depicted in Figure 2E–H. Animals immunized with vaccines prepared with Alhydrogel^®^ and Adju-Phos^®^ using 20 or 60 µg of chimeric protein exhibited reduced levels of IgM antibodies compared to all other antibodies. Both control (pre-immunization and adjuvant only) and experimental groups started with absorbances lower than 0.10 in the lowest serum dilutions (Figure 2E,F). Nevertheless, on day 31, the peak absorbances were 0.141 (Figure 2E), 0.135, and 0.159 (Figure 2F). The total IgM antibodies for 20 µg and 60 µg of CHIVAX 2.1 (samples at a dilution of 1:400) are shown in Figure 2G,H. There were no significant differences between the first and second immunizations in mice inoculated with Alhydrogel^®^ with 20 µg of CHIVAX 2.1; however, significant differences between the first and second immunizations using Adju-Phos^®^ with 20 µg of CHIVAX 2.1 (*p* ≤ 0.01) were observed (Figure 2G). Conversely, as shown in Figure 2H, significant differences at a 1:400 dilution for 21 and 31 days were recorded between the Alhydrogel^®^ group and the Adju-Phos^®^ group with 60 µg of protein. Serum samples from mice immunized solely with Alhydrogel^®^, Adju-Phos^®^, or those from the pre-immunization phase did not generate any antibodies.

**Figure 3:**
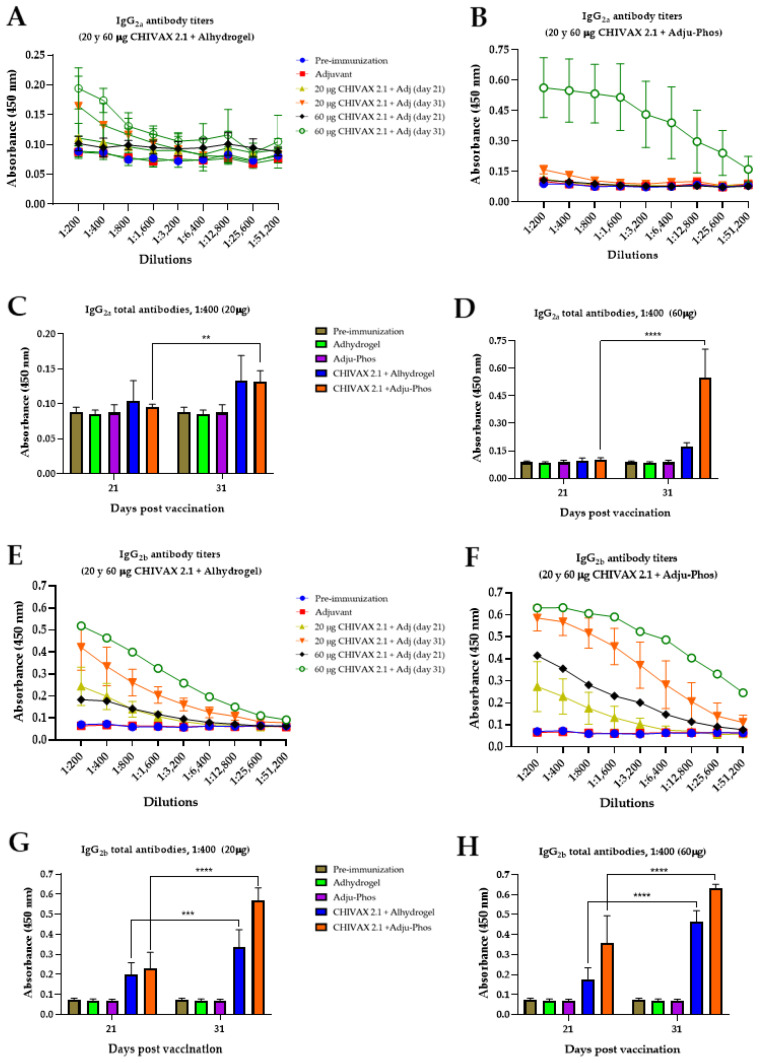
Serum IgG_2a_ and IgG_2b_ anti-CHIVAX 2.1 antibody production using two alum adjuvants with PHAD^®^. Animals were immunized with 20 or 60 µg of chimeric protein at 0 and 21 days. Titers for each bleeding are shown. (**A**) Specific IgG_2a_ antibody titers using Alhydrogel^®^. (**B**) Specific IgG_2a_ antibody titers with Adju-Phos^®^. A ratio of 1:400 dilutions for (**C**) IgG_2a_ for 20 µg vaccine and (**D**) IgG_2a_ for 60 µg vaccine. (**E**) Specific IgG_2b_ antibody titers using Alhydrogel^®^. (**F**) Specific IgG_2b_ antibody titers using Adju-Phos^®^. A ratio of 1:400 dilutions for (**G**) IgG_2a_ for 20 µg vaccine and (**H**) IgG_2b–f_ or 60 µg vaccine. Each result is the mean value of 5 animals ± SD. Unpaired *t*-test with Holm–Sidak’s comparison between the same groups of immunized animals for different postvaccine times (days 21 and 31). The differences were considered significant: *p* ≤ 0.001 (**), *p* ≤ 0.0001 (***), and *p* < 0.0001 (****).

Panels A and B in Figure 3 show the specific generation of the IgG_2a_ antibody with two formulations of CHIVAX 2.1. This result indicates that IgG_2a_ was present in mouse serum 10 days after the second antigen exposure (31 days). For the Alhydrogel^®^-vaccinated mice, absorbances of 0.1646 and 0.1941 were reached with 20 and 60 µg of CHIVAX 2.1, respectively (Figure 3A). In addition, Adju-Phos^®^ formulated with 20 and 60 µg of CHIVAX 2.1 induced maximum absorbance peaks of 0.1582 and 0.5621, respectively (Figure 3B). A single immunization with CHIVAX 2.1 was not sufficient for IgG_2a_ production in any group. No significant differences were found when Alhydrogel^®^ was used as an adjuvant, while the Adju-Phos^®^ concentration significantly increased between the first and second immunizations at 20 µg (** *p* ≤ 0.001) and 60 µg (**** *p* ≤ 0.0001) of CHIVAX 2.1 (Figure 3C,D). Figure 3E–H shows IgG_2b_ antibodies against CHIVAX 2.1. The IgG_2b_ response was evident from the first immunization; however, the maximum IgG_2b_ antibody levels were 0.421 and 0.519 for the Alhydrogel^®^ group and 0.585 and 0.631 for the Adju-Phos^®^ group for the two concentrations of CHIVAX 2.1 on day 31 (Figure 3E,F). Furthermore, significant differences were displayed following a full immunization scheme with both adjuvants and between different concentrations of CHIVAX 2.1 (Figure 3G,H).

**Figure 4:**
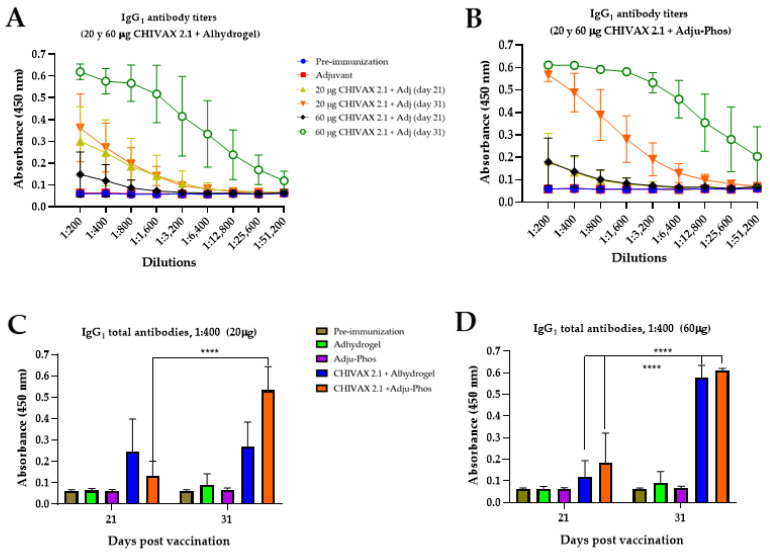
Serum IgG_1_ anti-CHIVAX 2.1 antibody production using two alum adjuvants with PHAD^®^. Animals were immunized two times (0 and 21 days). (**A**) Specific IgG_1_ antibody titers using Alhydrogel^®^. (**B**) Specific IgG_1_ antibody titers using Adju-Phos^®^. A ratio of 1:400 dilutions for (**C**) IgG_1f_ or 20 µg vaccine and (**D**) IgG_1f_ or 60 µg vaccine. Each result is the mean value of 5 animals ± SD. Unpaired *t*-test with Holm–Sidak’s comparison between the same groups of immunized animals for different postvaccine times (days 21 and 31). The differences were considered significant *p* < 0.0001 (****).

Finally, IgG_1_ antibodies were also evaluated (Figure 4A–D). Specific IgG_1_ antibodies were detected by the 21st day after the initial immunization with both adjuvants and 20 or 60 µg of CHIVAX 2.1. However, the IgG_1_ levels post second immunization (on the 31st day) were notably higher than those after the first immunization, with values of 0.361, 0.618 (Figure 4A), 0.568, and 0.611 (Figure 4B), respectively. The generation of IgG_1_ antibodies is directly proportional to the concentration of CHIVAX 2.1 in all groups during immunization for samples diluted at a ratio of 1:400 (Figure 4C,D).

**Figure 5:**
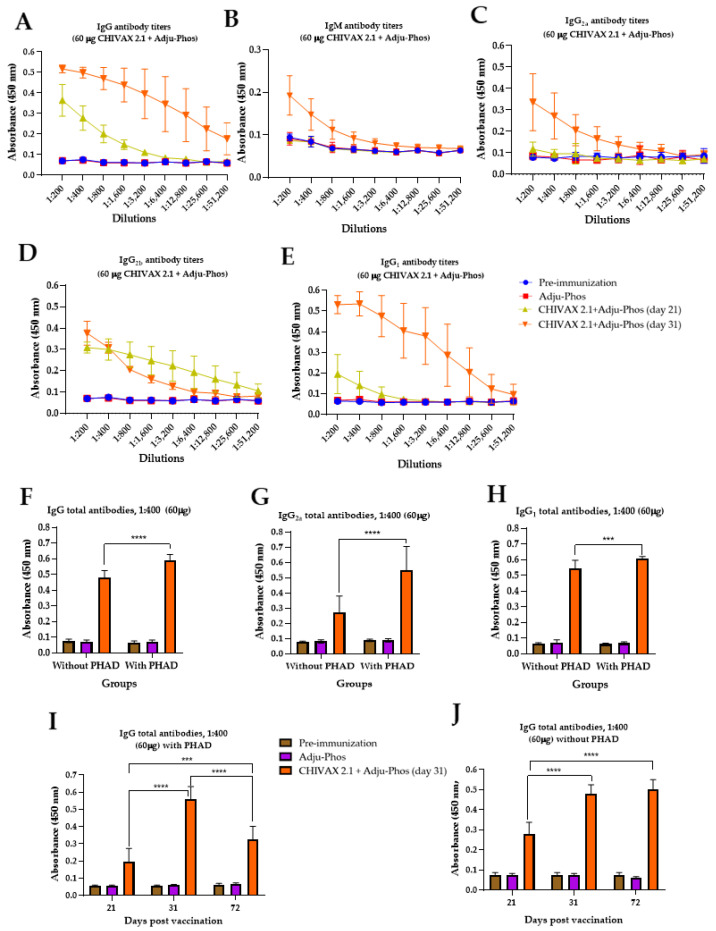
Anti-CHIVAX 2.1 serum antibody production using 60 µg of chimeric protein and Adju-Phos^®^ without PHAD^®^. Animals were immunized two times (0 and 21 days). (**A**) Specific IgG antibody titers. (**B**) IgM antibody titers. (**C**) IgG_2a_ antibody titers. (**D**) IgG_2b_ antibody titers. (**E**) IgG_1_ antibody titers. Each result is the mean value of 5 animals ± SD. A ratio of 1:400 dilutions for (**F**) IgG, (**G**) IgG_2a_ and (**H**) IgG_1_ for vaccines without PHAD^®^ and with PHAD^®^. Total specific IgG antibodies (**I**) with PHAD^®^, (**J**) without PHAD^®^ until 72 days post immunization. Unpaired *t*-test with Holm–Sidak’s comparison between the same groups of immunized animals for different postvaccine times (days 21, 31, and 72). The differences were considered significant *p* ≤ 0.0001 (***), and *p* < 0.0001 (****).

Vaccines formulated with 60 µg of the recombinant protein CHIVAX 2.1 and Adju-Phos^®^ yielded the best humoral response (IgG, IgM, IgG_2a_, IgG_2b_, and IgG_1_) compared to other experimental groups. It is well known that the generation of antibody subclasses is specific to the antigen and the adjuvants used, which induce either a T_H1_- or T_H2_-biased response. T_H1_ cells are identified by the production of mouse IgG_2a_/IgG_2b_ and IgG3 antibody subclasses [4,29]. So far, experiments have been performed with vaccines formulated with PHAD^®^ and aluminum adjuvants. However, we decided to assess the efficacy of vaccines devoid of PHAD^®^, aiming to determine whether the increased IgG and IgG_2a_ antibody production resulted from adding PHAD^®^ to the chimeric vaccine and alum adjuvant. The generation of specific antibodies for vaccines minus PHAD^®^ is observed in Figure 5A–H.

A considerable rise was observed between the first and second immunizations (on days 21 and 31) for all tested antibodies, except for the IgG_2b_ antibody (Figure 5D). The production of IgG and IgM increased by 0.5166 and 0.1932, respectively (Figure 5A and 5B). Similarly, the IgG_2a_ antibody level was 0.336 at the second immunization (Figure 5C). IgG_1_ immunoglobulins reached an antibody titer of 0.5314 on day 31 post immunization (Figure 5E).

High serum IgG_1_ antibody titers are closely tied to an increase in RBD-specific neutralizing IgG and the generation of memory B-cells, particularly for vaccines formulated with an emulsion-based adjuvant [3,30]. A significant difference was noted in IgG (*p* ≤ 0.0001), IgG_2a_ (*p* ≤ 0.0001), and IgG_1_ (*p* ≤ 0.001) antibody production with vaccines formulated with and without PHAD^®^ after all immunization schemes (31 days) (Figure 5F–H).

Approximately, a ∼2-fold increase was observed in total IgG_2a_ antibody at a 1:400 serum dilution with and without PHAD^®^ (0.548 and 0.272, respectively), suggesting an activated cellular response leaning toward a T_H1_-subtype balance, ideal for protecting against viral diseases [31].

Additionally, specific-IgG (1:400 dilution) for the 60 µg CHIVAX 2.1 vaccine with and without PHAD^®^ up to 72 days post immunization is displayed in Figure 5I,J. The formulations with PHAD^®^ demonstrated a high level of 0.561 at 31 days, which significantly decreased to 0.323 by day 72, with marked differences noted between days 21, 31, and 72 (Figure 5I). In contrast, the vaccines lacking PHAD^®^ recorded the lowest levels at 31 days (0.481) and this remained relatively stable through day 72 (0.499). No significant difference was observed between the measurements on day 31 and day 72 (Figure 5J). The findings of this study imply that the concentration of CHIVAX 2.1 and the type of aluminum adjuvants used in vaccines substantially affect specific antibody titers and the production of the immunoglobulin subclass

### 3.2. Neutralizing Activity Evaluated by Surrogate Virus Neutralization Assay

**Figure 6:**
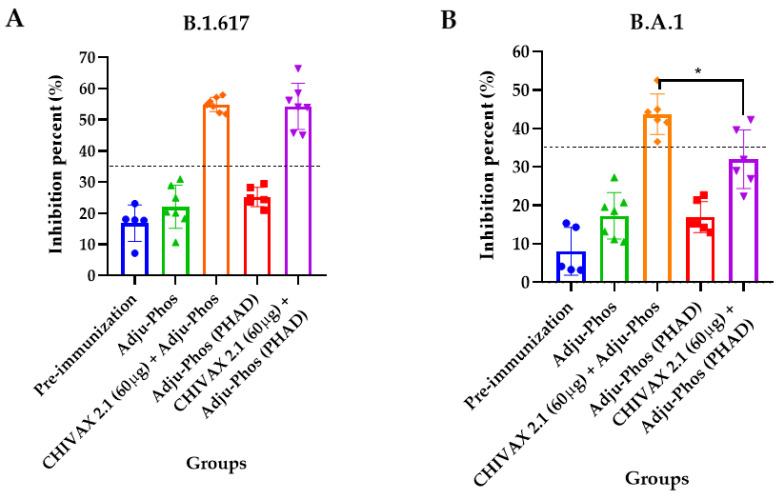
Neutralizing antibodies against different variants of SARS-CoV-2. Inhibition percent of immunized mice serum with 60 µg of CHIVAX 2.1 with and without PHAD^®^ against (**A**) Delta B.1.617.2, (**B**) Omicron B.A.1 variants were determined at 450 nm. The results are expressed as the mean ± SD of five independent experiments in duplicate. Each result represents the average of the animal’s data. The asterisk expresses significant differences, *p* < 0.05 (*), by unpaired *t*-student test for each pair of data between two vaccine formulations. The threshold was 30% inhibition.

Figure 6 presents results for animals immunized with 60 µg of recombinant protein (CHIVAX 2.1), with or without PHAD^®^. Neutralizing antibodies against the SARS-CoV-2 Delta variant were produced. In the sera of mice vaccinated using a vaccine formulation without PHAD^®^, a 55% inhibition was observed. In contrast, a 54% inhibition was detected in the sera of mice immunized with formulations containing PHAD^®^ (Figure 6A). Regarding the Omicron variant, only the sera from mice vaccinated with formulations without PHAD^®^ showed a 42% inhibition of RBD-ACE_2_ binding. The percentage of neutralization reached 32% using the sera from mice immunized with the vaccine formulated with PHAD^®^ (Figure 6B). All the pre-immunization sera and adjuvant concentrations were below the 35% threshold for inhibition for both variants.

### 3.3. Cellular Response Against CHIVAX 2.1

**Figure 7:**
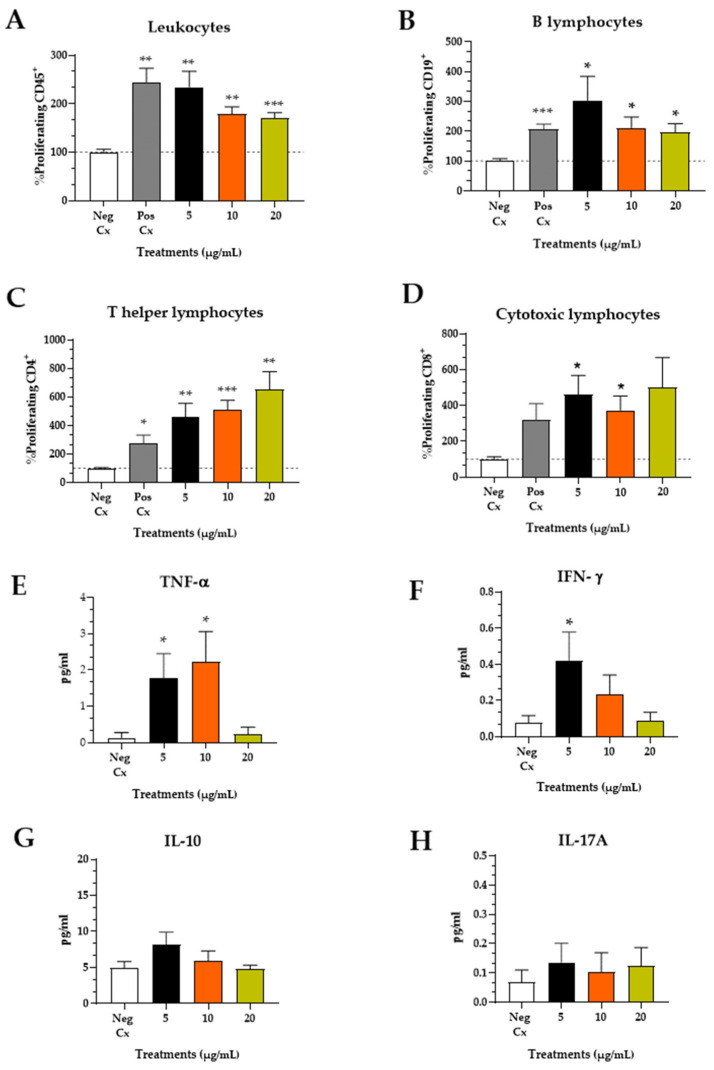
In vitro cellular stimulation with CHIVAX 2.1. Proliferation of splenocytes from a full scheme of immunization mice with CHIVAX 2.1 (60 µg), + Adju-Phos^®^ + PHAD^®^ after 109 days. Cells were obtained after 72 h of stimulation. (**A**) Leukocytes, (**B**) B lymphocytes, (**C**) helper T lymphocytes, and (**D**) cytotoxic lymphocytes. Cytokines from the media supernatant 20 h after stimulation (**E**) TNF-α, (**F**) IFN-γ, (**G**) IL-10, (**H**) IL-17A. Mann–Whitney test for multiple comparisons was used to compare treatments. The proportions of negative control and treated cells and, for the same comparison, cytokines in the media supernatant were also analyzed by unpaired *t*-test. Data shown as mean ± SEM of 3 different animals by duplicate (*p* ≤ 0.05 (*), *p* ≤ 0.01 (**), *p* ≤ 0.001 (***)).

Determining the phenotype of the cellular response and the cytokine profile that induces antibody responses to a virus is essential for assessing the effectiveness of a vaccine against SARS-CoV-2 [18]. The production of antibodies is directly correlated with the activation of CD4+ T cells. After the second immunization, specific IgG antibody levels significantly varied for the animals immunized with Adju-Phos^®^ and 60 µg of protein + PHAD^®^. Since a long-term vaccine reaction is a critical feature of successful immunization, we evaluated the proliferation-inducing capacity of CHIVAX 2.1 in immune cells from the animals’ spleens using the complete scheme with CHIVAX 2.1 (Figure 7A–D). The vaccination of C57BL6 mice with CHIVAX 2.1 triggered an antigen-specific CD4+ T-cell response. A significant CD8^+^ T-cell response also was observed when the cells were treated with 5 and 10 µg of the chimera protein.

Leukocytes stimulated with 1.5 µg/mL CHIVAX 2.1 exhibited a similar proliferation level to ConA (*p* = 0.0057 **). However, leukocyte proliferation was inversely proportional to the concentration of the chimeric protein (Figure 7A), suggesting an overstimulation effect. Notably, a lower concentration of 5 µg/mL induced B-cell (*p* = 0.0445 *) and T cytotoxic lymphocyte proliferation (*p* = 0.0145 *) compared to the negative control, while higher concentrations (10 and 20 µg/mL) had less of an effect (Figure 7B,D). Contrary to the responses of general leukocytes, B-cells, and T cytotoxic cells to increased antigen concentrations, the proliferation of T helper cells was directly proportional to CHIVAX 2.1 (5 µg/mL *p* < 0.01; 10 µg/mL *p* < 0.001; 20 µg/mL *p* < 0.01). This outcome is interesting because it implies that the function of these cells may not be diminished by antigen overstimulation (Figure 7C).

Concerning the soluble factors produced by splenocytes, TNF-α production (Figure 7E) was induced by both low (*p* = 0.0313 *) and medium (*p* = 0.0286 *) antigen concentrations but not by the higher (*p* = 0.6942) concentration. A similar pattern was observed for IFN-γ production (Figure 7F), where only the low dose was significantly higher than the unstimulated control (*p* = 0.0472 *). Interestingly, IL-10 production (Figure 7G), a regulatory cytokine, showed no significant difference from the negative control at any concentration. This was consistent with the levels of IL-17 in the medium supernatant (Figure 7H). A cluster of differentiated CD4+ TH1 cells is known to produce IL-2, TNF-α, TNF-β, and IFN-γ, whereas CD4^+^ TH2 cells are characterized by their production of IL-4, IL-5, IL-6, and IL-13 [18,32]. Therefore, 78 days after the last immunization, splenic leukocytes could proliferate after 72 h of exposure to a low concentration (5 µg/mL) of CHIVAX 2.1. Additionally, B lymphocytes responded to a low antigen concentration, suggesting that a humoral response can be induced, along with the anticipated expansion of T helper cells.

## 4. Discussion

In this study, we demonstrated the induction of specific antibody titers for IgG, IgM, IgG_2a_, IgG_2b_, and IgG_1_ in diluted sera (1:200) from mice immunized with CHIVAX 2.1 (60 µg) in Adju-Phos^®^ and PHAD^®^. Aluminum phosphate is an amorphous suspension; depending on its production conditions, particle size, structure, and morphology, it can exhibit surface phosphate groups and can be better absorbed by water molecules, consequently enhancing the immunological potentiation compared to other aluminum adjuvants [15,33,34]. Our results align with other vaccines evaluated in mice, which have shown that concentrations of IgM are either too low or undetectable and are not linked to the cellular response [16,35]. Moreover, significant variations in the concentrations of IgG, IgG_1_, and IgG_2a_ were observed between formulations with and without PHAD^®^. The mechanism of monophosphoryl lipid A (PHAD^®^) is not fully understood. However, lipophilic adjuvants are structured with hydrophilic head groups and hydrophobic acyl chains that tend to aggregate as liposomes in aqueous environments, making them ideal antigen-delivery vehicles [16,36]. It is common for chimeric recombinant vaccines to use alum adjuvants along with accessory molecules like PHAD^®^ or adjuvants containing these components as exemplified by AS04 or AS03, a squalene-based system. These enhance T helper cell responses by promoting TRIF signaling, actively inhibiting TLR4 and thus mitigating the limitations of these formulations, a practice also observed in coronavirus vaccines [29,30,37].

We have demonstrated that CHIVAX 2.1 neutralizes two VoCs through cross-neutralizing activity mediated by RBD-specific IgG antibodies. Four amino acid modifications in the RBD, particularly within the S subunit of the spike protein, have been the focus of studies that assess their impact on virulence and immune evasion. These include mutations in N501Y, common to Alpha, Beta, Gamma, and Omicron; E484K/Q/A and K417T/N, present in Beta, Gamma, and Omicron; and L452R, exclusive to the Delta lineage [38,39,40]. A key advantage of chimeric vaccines is the incorporation of one or more amino acid substitutions into selected epitopes, which can increase vaccine immunogenicity and protect against multiple virus variants [9,10,12]. The use of short peptides containing B- and T-cell epitopes to formulate the chimeric protein might seem disadvantageous if targeting the RBD region exclusively. However, as earlier studies have shown [41], the virus uses only the RBD to adhere to its receptor, the ACE_2_ molecule on human cells, and it has been proven that antibodies delivered to this region alone can prompt neutralization. Incorporating peptides with key epitopes of new variants, this platform could generate both cellular and humoral protective immune responses against multiple VOCs in one molecule. The robust activation of CD4^+^ and CD8^+^ T cells and the secretion of TNF-α, IFN-γ, and IL-17A was evident. Even though IL-10 indicated a non-significant rise with a lower CHIVAX 2.1 concentration after 20 h of exposure, this early response was not related to the increased antigen concentration. This might actually be beneficial in avoiding the induction of tolerantly exhausted lymphocytes or due to a booster effect. Polarization towards TH1 and TH17 CD4^+^ T cells induced by the combination of aluminum adjuvants is revealed by the production of IgG_2a_ and IFN-γ [1,23,24]. TLR9 agonists like CpG have also been added to alum vaccine formulations for the induction of TH1 CD4^+^ T cells by a mechanism dependent on the Toll-like receptor 9-mediated MyD88 activation in dendritic cells [1,3,13].

Chimeric vaccines composed of protein, mRNA, and VLP, engineered with multiple conserved epitopes of the SARS-CoV-2 spike protein, activate systemic and local humoral responses. They also elicit specific IgG neutralizing antibodies against SARS-CoV-2 through the activation of CD4^+^ B-cells via the TLR signaling mechanism, which triggers cytokine production [9,10,12,42]. These vaccines present a viable alternative against a broad spectrum of viral infections that have high epidemiological significance, including zoonotic infections [11].

The chimeric CHIVAX 2.1 protein, composed of B- and T-cell epitopes from the RBD of the S protein of various SARS-CoV-2 VoCs, demonstrated antigenic and immunological effectiveness in animals that received two doses, preferably mainly with 60 µg vaccine formulations, Adju-Phos, and PHAD^®^. The production of specific antibodies (IgG, IgG_1_, and IgG_2a_) was more substantial after two immunization doses than after a single one, with IgG-specific antibodies detected at 72 days, indicating memory B-cell activation. Furthermore, the generated neutralizing antibody IgG RBD displayed cross-reactivity to VoCs. Our vaccination scheme demonstrated long-term effectiveness 72 days post first immunization. Leukocytes, as well as B- and T-cells, proliferated significantly with lower antigen stimulation, accompanied by the production of proinflammatory and antiviral response cytokines, such as TNF-α and IFN-γ. This implies that after a complete two-month vaccination scheme, the immune system is primed to respond to SARS-CoV-2 antigens and initiate a humoral and cytotoxic response. While further characterization of the memory immune response is crucial, these findings suggest that this immunization scheme successfully prompts a sustained response capable of quickly reacting to the virus components.

These findings are promising for multi-epitope vaccine candidates in the context of viral infections. Exposure to CHIVAX 2.1 led to a cytotoxic response, which was accompanied by an increase in IFN-γ. This could potentially be beneficial for an antiviral response, predominating in T_H1_ cells rather than in T_H2_ cells, or it could balance the T_H1_:T_H2_ ratio through the use of mixed alum adjuvants.

## Data Availability

The original contributions presented in the study are included in the article, further inquiries can be directed to the corresponding authors.

## Supplementary Materials

The following supporting information can be downloaded at: https://www.mdpi.com/article/10.3390/pathogens13121081/s1, Figure S1: Representative gating path strategy.
