# Supplementary material for: An Evaluation of the Cellular and Humoral Response of a Multi-Epitope Vaccine Candidate Against COVID-19 with Different Alum Adjuvants
Source: Pathogens. 2024 Dec 9;13(12):1081. doi: 10.3390/pathogens13121081 (PMC11728595; doi:10.3390/pathogens13121081)
Supplement: Supplementary file 1 [file pathogens-13-01081-s001.zip › pathogens-3288572-supplementary.pdf]

Material suplementary

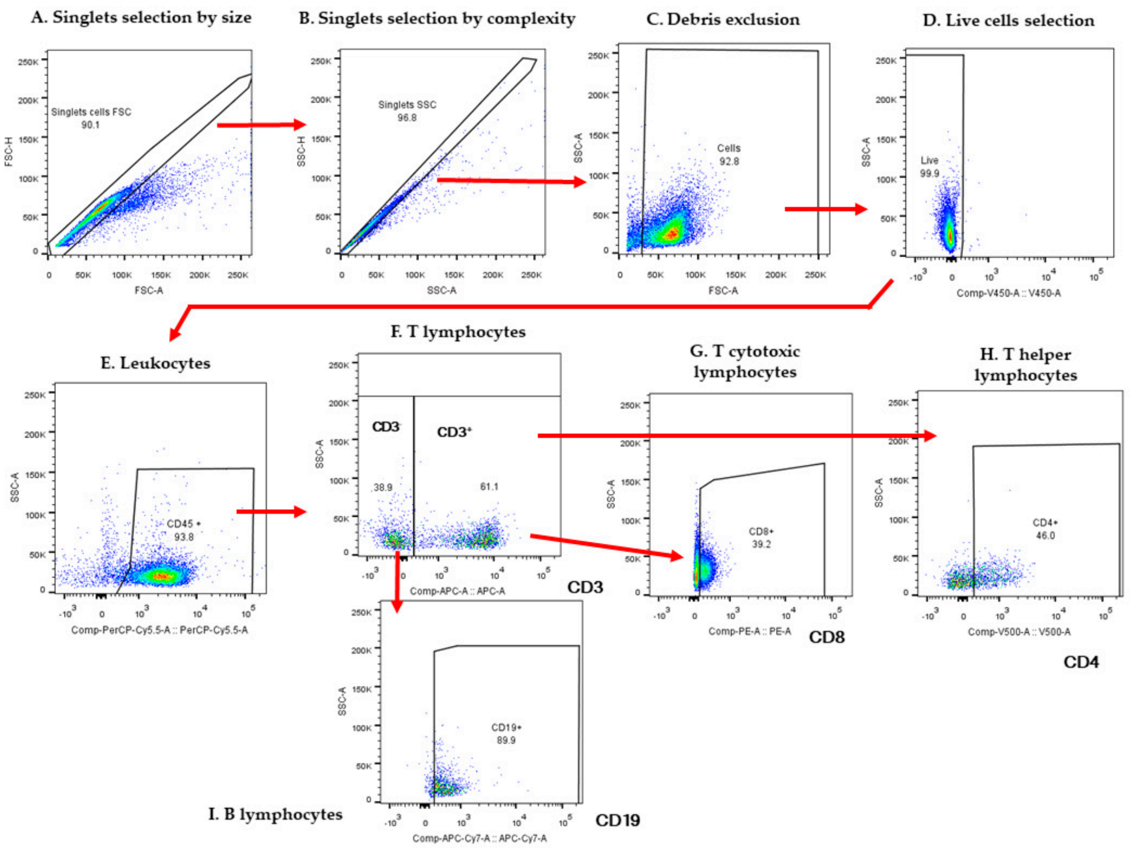

Supplementary Figure S1**.** Representative gating path strategy. Flow cytometry analysis of splenocytes cultured for 72 hours. A., B. Double single-cell selection by size (A) and complexity (B). C. *Debris* exclusion. D. Live cells were gated as negative events for the exclusion viability dye Zombie Violet. E. From live cells, leukocytes were gated as CD45+. F. T lymphocytes ($CD3^+$) obtained from $CD45^+$ leukocytes. G. Cytotoxic T lymphocytes from $CD3^+$ cells. H. T helper lymphocytes from CD3+ events. I. B lymphocytes ($CD19^+$) obtained from CD3- cells.
